# Correlation between Non-Polio Acute Flaccid Paralysis Rates with Pulse Polio Frequency in India

**DOI:** 10.3390/ijerph15081755

**Published:** 2018-08-15

**Authors:** Rachana Dhiman, Sandeep C. Prakash, V. Sreenivas, Jacob Puliyel

**Affiliations:** 1 Department of Pediatrics, St Stephens Hospital, Delhi 110054, India; dhimanrachna48@gmail.com (R.D.); sandeepcpp25@gmail.com (S.C.P.); 2 Department of Biostatistics, All India Institute of Medical Sciences, New Delhi 10029, India; sreevishnubhatla@gmail.com

**Keywords:** polio surveillance, AFP, oral polio vaccine, non-polio acute flaccid paralysis, Guillain Barre Syndrome

## Abstract

The last case of polio from India was reported in 2011. That year, the non-polio acute flaccid paralysis (NPAFP) rate in India was 13.35/100,000, where the expected rate is 1–2/100,000. A previous study of data from 2000 to 2010 has detailed the NPAFP rate in a state correlated with the pulse polio rounds conducted there, and the strongest correlation with the NPAFP rate was found when the number of doses from the previous 4 years were used. However, a simple association being found with regression analysis does not prove a causal relationship. After publication of those findings, as the threat of polio had lessened, the number of rounds of OPV administration was brought down. The present study has been done to look at data till the end of 2017, to see if the incidence of NPAFP declined with this reduction in polio immunization rounds. We used polio surveillance data acquired by the Government of India from 2000–2017. Correlation of the NAFP rate to the number of polio rounds in the state was examined, and the cumulative effect of polio doses administered in previous years was sought. NPAFP rate correlated with the OPV pulse polio rounds in that year (*R* = 0.46; *p* < 0.001), and the NPAFP rate started to decrease from 2012 when the number of pulse polio rounds had decreased. NPAFP rates in the states of Uttar Pradesh (UP) and Bihar were the highest in the country. Looking at the high-NPAFP states of UP and Bihar, we found that the correlation coefficient was strongest when doses used over 5 years was considered (*R* = 0.76; *p* < 0.001). The response to the reduction in OPV rounds (de-challenging) adds credence to the assumption that OPV was responsible for the change in the NPAFP rate. Now that India has been polio-free for over 6 years, we propose that we may be able to reduce NPAFP by further reducing pulse polio rounds.

## 1. Introduction

Surveillance of polio is complicated, due to the fact that 99% of those infected do not exhibit paralysis [1]. Given this obscure presentation of polio, it is vital that all cases of acute flaccid paralysis (AFP) are studied to ensure that they are successfully surveilled. In India, active poliomyelitis and AFP surveillance began in 1997 [2].

For polio surveillance purposes, a diagnosis of AFP is defined as any patient <15 years of age with acute onset flaccid paralysis, or a patient of any age in whom a clinician suspects polio [3]. Those with other, more obvious causes (like trauma) are excluded [4]. Short-lived paralysis, as with Todd’s paresis, are excluded [5]. Marx et al. have reviewed and listed the causes of non-polio AFP [6]. Stool specimen testing was used to try and separate the true cases of polio AFP from that of non-polio AFP (NPAFP) [3]. It was expected that conducting surveillance in this way would help identify reservoirs of wild poliovirus transmission and provide evidence that wild polio transmission was not occurring [7].

AFP surveillance data from America shows that there were 1975 cases of AFP reported in 2017, corresponding to an AFP rate of 1.17 cases per a population of 100,000 [8]. Internationally, the incidence of NPAFP is 1 to 2/100,000 in the under-15 population [6,9]. In the absence of wild polio transmission, it was expected that the AFP rate would reduce to around 2/100,000, which is considered an acceptable NPAFP rate [10].

The surveillance quality indicators from India show that surveillance has been exemplary, and the last case of polio reported was in 2011 [11]. However, the anticipated fall in the AFP rate to 2 per 100,000 has not yet materialized.

Analysis of data over 10 years (from 2000 to 2010) showed that the NPAFP rate increased nationally during this time [12]. The NPAFP rate in 2010 was 12/100,000, which was some way away from the expected number of 2/100,000. It has been reported that in 2005 there was a sharp increase in the national NPAFP rate, which coincided with the introduction of a high-potency monovalent vaccine that contained 5 times the number of Type 1 viruses, compared to that contained in the previously used vaccine [13]. The NPAFP rate, which was 3.11/100,000 in 2004, more than doubled (to 6.43/100,000) in 2005.

Some states had a higher rate of NPAFP than others. In 2011, the NPAFP rate in UP and Bihar was 25/100,000 and 35/100,000 respectively.

Pulse polio immunisation refers to periodically vaccinating all children under the age of five years against the polio virus (in a defined region) for the purposes of eliminating the virus. The NPAFP rate in the states over the years was examined, and it was found that the number of pulse polio rounds conducted had a high correlation with the NPAFP rate in the state. There was no association with other socioeconomic factors of the state, such as literacy levels, population density, or income per capita [12]. In one of the years (2011) there were an additional 47,500 children with paralysis [12] which was over and above the assumed NPAFP rate of 2/100,000 [6,9].

It has been suggested that the rise in the recorded rates of NPAFP was an artifact related to over-enthusiastic reporting promoted by the government’s efforts to improve surveillance [14]. An analysis in 2005 showed that where one-fifth of the cases of NPAFP were followed up at 60 days (in the state of UP), 8.5% of them had died, and 35% were found to have been left with residual paralysis [15]. Sathyamala analysis of NPAFP data from UP found that the mortality rate in patients with NPAFP was twice the mortality rate for wild polio [16]. This suggests that the recorded cases of NPAFP were not just instances of exaggerated reporting.

It is crucial to note that a mere association with regression analyses does not prove a causal relationship. Aggregated variables examining cross-sectional data which have no bearing on what happens to individuals can result in ecological fallacies, and necessitates more in-depth analyses. De-challenging after challenging is one way to test for a causative relationship. In de-challenging, the suspected offending agent is withdrawn, or its dose reduced, which should result in the amelioration of the adverse event [17].

After the publication of the findings reported above [12], because the threat of polio had receded, the number of polio doses administered to children each year was gradually reduced from 2012. The present analysis was done looking at present data till 31 December 2017, to see whether the reduction in the number of doses of OPV administered in recent years was associated with a decline in the reported NPAFP rates. This would add strength to the likelihood of a causative association.

## 2. Materials and Methods

Data used was obtained from the National Polio Surveillance Programme (NPSP), reported by the Government of India. Web pages that were no longer available on the internet were accessed using the Wayback machine website (https://web.archive.org/web/20010201070500/http://npspindia.org:80/ and related pages). The data was mostly complete, except for the year 2003 where data after October 2003 was not accessible. Data on the number of people with NPAFP and the NPAFP rate from each state per year from 2000 till 31 December 2017 was extracted, and is available online (https://bit.ly/2z3ywUY). The number of pulse polio immunization rounds in the state was also recorded for each year, as was done in our earlier analysis [12]. Data from all 36 states and Union Territories provided in the NPSP data sheets were included. If different doses were employed in different areas of the state, the mean value was used in the analysis. Regression analysis was completed, and the NPAFP rate was the outcome of interest, with pulse polio rounds being considered the explanatory variable. As in the previous analysis, here we also explored whether the NPFP rate was related to the number of pulse polio rounds deployed in the state. We did further analysis by looking at the cumulative effect of adding doses from the previous years. The total rate of NPAFP for a certain year was also compared to the expected numbers for that year, to see how different the actual number was to the expected rate.

## 3. Results

As more pulse polio rounds were conducted the NPAFP rate was found to increase during the period of 2000–2011, but began to decrease from 2012. The Pearson correlation was found to be statistically significant (Regression Coefficient *R*) = 0.46; *p* Value (*p*) < 0.001), and regression analysis suggested that the NPAFP rate increased by 1.4 for every round of pulse polio (95% CI: 1.2–1.6). In other words, for each round of pulse polio there was an increase of 1.4 cases of NPAFP per under-15 population of 100,000.

The highest NPAFP rates were seen in Uttar Pradesh and Bihar, where there was an increase of 2.7 cases of NPAFP per under-15 population of 100,000 for each round of pulse polio (CI 1.1–4.2). *R* was equal to 0.52 (*p* < 0.001). The NPAFP rate had the highest correlation level with the cumulative doses in the previous 5 years (*R* = 0.76; *p* < 0.0001). Table 1 outlines this improvement in correlation level when the cumulative doses from the previous five years is considered.

**Figure 1:**
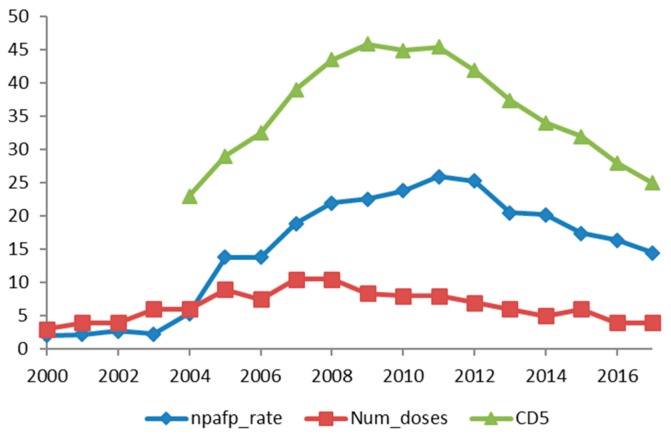
Non-polio AFP over the years in the state of UP alongside the 5-year cumulative doses of OPV. npafp_rate: Non-polio acute flaccid paralysis rate; Num_doses: Number of pulse polio rounds; CD5: Cumulative doses in the past 5 years.

**Figure 2:**
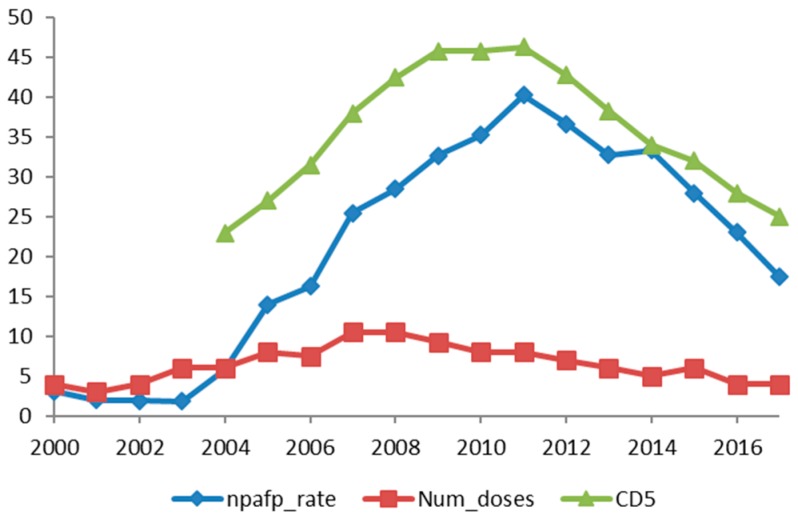
Non-polio AFP over the years in the state of Bihar alongside the 5-year cumulative doses of OPV. npafp_rate: Non-polio acute flaccid paralysis rate; Num_doses: Number of pulse polio rounds; CD5: Cumulative doses in the past 5 years.

Figure 1 and Figure 2 show the non-polio AFP over the years in the states of UP and Bihar alongside the 5-year cumulative OPV doses. The fall in NPAFP rate for the first time in 2012 with a decrease in the OPV doses is illustrated.

We calculated the number of paralyzed children each year which exceeded the expected numbers (assuming a NPAFP rate of 2/100,000) and the results are displayed in Table 2. A total of 640,000 children developed NPAFP in the years 2000–2017, suggesting that there were an additional 491,000 paralyzed children above our expected numbers for children with NPAFP.

## 4. Discussion

From the results, the NPAFP rate has been shown to decline with a reduction in the pulse polio doses. This response to de-challenging adds weight to the likelihood of there being a causative association with OPV vaccinations.

Studies from Finland have shown an association of Guillain Barre Syndrome (GBS) with OPV vaccination campaigns [18,19], and similar findings have been reported from Turkey [20]. It should be noted that in the study by Kinnenen, the diagnosis of GBS was made by using consistent criteria throughout the observation period. The Institute of Medicine (vaccine safety committee) has suggested that it is biologically plausible that OPV causes GBS and that the evidence favors the existence of a causal relationship [21]. Adverse reactions to OPV is said to cause demyelination, including GBS, within 5 days to 6 weeks of vaccinations. The risk difference is approximately 2.5 per 100,000 people during that window period [21]. This is not very different from the increase of 2.7 cases of NPAFP /100,000 population, per pulse polio round that we found in UP and Bihar. The NPAFP rate could go up to 25/100,000 if 10 rounds of pulse polio are used in the year, as we saw in UP and Bihar.

The AFP surveillance data from India does not provide information about the etiology of NPAFP, and it is tempting to suggest that OPV-related GBS may be the cause for the rise in NPAFP seen across the country.

However, GBS is unlikely to be the sole reason for the increase in NPAFP. GBS, caused by OPV, was usually reported soon after an OPV campaign [18,19,20]. Our finding that the NPAFP rate in each year correlated best with the cumulative doses administered over the previous 5 years, suggests that they may not all have been OPV-caused GBS (occurring in 5 days to 6 weeks).

We have previously shown that the NPAFP rate increases sharply when more than six rounds of pulse polio are used in the year [22]. We speculated that repeated doses of the live vaccine virus delivered to the intestine may colonize the gut and alter the viral microbiome of the intestine, and this can result in strain shifts of enteropathogens. It is possible that new neurotropic enteroviruses colonizing the gut may induce paralysis.

Although more studies are needed to confirm a causal association, the dose response to de-challenges makes a causal association more likely. A rapid epidemiological appraisal can be done in a national case-control study, where exposure to multiple doses of OPV can be examined in children with NPAFP against healthy controls.

While the mechanism involved is speculative, our findings suggest that the increase in NPAFP (and the later decrease in such cases) was indeed an adverse effect of the pulse polio immunization programme. This increase in NPAFP was noticed only because of excellent surveillance methods, the meticulous recording of data, accurate pooling of the figures nationally and making it available in the public domain—the 491,000 additional cases of paralysis would not have been noticed otherwise. From the public health point of view, this underlines the importance of collecting and reporting aggregated adverse events following immunization (AEFI) data which is essential to making our immunization programmes safer.

## 5. Conclusions

The polio eradication programme succeeded in drastically reducing the global spread of this disease, which was achieved through the use of immunization with OPV. While commending this enormous effort at eradication, our observation supports the hypothesis that the frequency of pulse polio administration is directly or indirectly related to the incidence of NPAFP. It is hoped that this finding will help continue efforts at optimizing the dose schedule of OPV administration and result in a reduction in NPAFP—which is a feasible hope, as the incidence of wild polio is currently at an all-time low.

## Figures and Tables

**Table 1:** Correlation of non-polio acute flaccid paralysis (NPAFP) rate to cumulative doses of OPV employed over six years in Uttar Pradesh (UP) and Bihar.

Serial No.	Number of Years of Cumulative Doses	NPAFP Rate Regression Coefficient *R*	*p* Value
1	1 Year	0.52	*p* < 0.001
2	2 Years	0.60	*p* < 0.001
3	3 Years	0.67	*p* < 0.001
4	4 Years	0.72	*p* < 0.001
5	5 Years	0.76	*p* < 0.001
6	6 Years	0.75	*p* < 0.001

**Table 2:** Excess NPAFP in each year from 2000–2017.

Year	AFP Rate	NPAFP Rate	AFP	NPAFP	Expected NPAFP	Excess NPAFP
2000	2.16	1.94		7260	7485	−225
2001	1.91	1.75	7510	6858	7838	−980
2002	2.45	1.87	9713	7404	7919	−515
October 2003	2.11	1.67	6850	5417	6487	−1070
2004	3.24	3.11	13,274	12,765	8209	4556
2005	6.54	6.43	27,049	26,586	8269	18,317
2006	7.63	7.35	32,194	31,024	8442	22,582
2007	9.71	9.32	41,534	39,831	8547	31,284
2008	10.5	9.93	45,586	43,103	8681	34,422
2008	11.64	11.33	50,412	49,082	8664	40,418
2010	12.7	12.65	55,785	55,548	8782	46,766
2011	13.55	13.35	60,750	59,849	8966	50,883
2012	13.97	13.61	61,038	59,462	8738	50,724
2013	12.51	12.48	54,645	54,511	8736	45,775
2014	12.48	12.48	53,933	53,910	8639	45,271
2015	10.78	10.77	46,970	46,957	8720	38,237
2016	10.61	10.16	46,524	44,571	8774	35,797
2017	8.97	8.72	39,339	38,232	8769	29,463
Totals			653,106	642,370	150,666	49,1704
